# Electrical Impedance of Surface Modified Porous Titanium Implants with Femtosecond Laser

**DOI:** 10.3390/ma15020461

**Published:** 2022-01-08

**Authors:** Paula Navarro, Alberto Olmo, Mercè Giner, Marleny Rodríguez-Albelo, Ángel Rodríguez, Yadir Torres

**Affiliations:** 1 Departamento de Tecnología Electrónica, Escuela Técnica Superior de Ingeniería Informática, Universidad de Sevilla, Av. Reina Mercedes s/n, 41012 Sevilla, Spain; paunavgon2296@gmail.com; 2 Departamento de Ingeniería y Ciencia de los Materiales y del Transporte, Escuela Politécnica Superior, Calle Virgen de África 7, 41011 Seville, Spain; lralbelo@us.es (M.R.-A.); ytorres@us.es (Y.T.); 3 Instituto de Microelectrónica de Sevilla, IMSE-CNM (CSIC, Universidad de Sevilla), Av. Américo Vespucio s/n, 41092 Sevilla, Spain; 4 Departamento de Citología e Histología Normal y Patológica, Universidad de Sevilla, Av. Doctor Fedriani s/n, 41009 Sevilla, Spain; mginer@us.es; 5 Escuela Politécnica Superior, Universidad da Coruña, Calle Mendizábal s/n, 15403 Ferrol, Spain; angel.rcarballo@udc.es

**Keywords:** cell culture, electrical impedance, femtosecond laser, osseointegration, porous titanium

## Abstract

The chemical composition and surface topography of titanium implants are essential to improve implant osseointegration. The present work studies a non-invasive alternative of electrical impedance spectroscopy for the characterization of the macroporosity inherent to the manufacturing process and the effect of the surface treatment with femtosecond laser of titanium discs. Osteoblasts cell culture growths on the titanium surfaces of the laser-treated discs were also studied with this method. The measurements obtained showed that the femtosecond laser treatment of the samples and cell culture produced a significant increase (around 50%) in the absolute value of the electrical impedance module, which could be characterized in a wide range of frequencies (being more relevant at 500 MHz). Results have revealed the potential of this measurement technique, in terms of advantages, in comparison to tiresome and expensive techniques, allowing semi-quantitatively relating impedance measurements to porosity content, as well as detecting the effect of surface modification, generated by laser treatment and cell culture.

## 1. Introduction

In recent decades, due to the aging of the population and change in lifestyles, millions of people have been affected by orthopedic, oral, and maxillofacial diseases [1]. Bone tissues are also exposed to damage due to degenerative or traumatic diseases that can cause serious disabilities and, therefore, carry high economic and social costs [2]. Biomaterials is one promising solution to solve such problems, as it can be used to manufacture medical devices for replacement of human tissues, such as teeth, bones, and cartilages. In addition, the demand for biomaterials is dramatically growing due to increasing maturity of materials manufacturing technologies [1,2].

Titanium and its alloys are considered one of the best choices for modern metallic implants, owing to their excellent biomechanical compatibility, long-term stability, and corrosion resistance in biological surroundings [3,4]. However, vital issues, such as bone resorption of tissues adjacent to the implant, related to the stress shielding phenomenon [5] and poor osseointegration, caused by bacteria proliferation or implant loosening, are still challenging problems to solve.

Two main approaches have been established to lessen or eliminate the stress shielding phenomenon on titanium implants. The first potential way is the use of β-titanium alloys with elements of low toxicity (Nb, Ta, Mo, and Zr) [6]. However, the use of porous titanium could be a more economical route for manufacturing titanium implants, with a stiffness and yield strength close to cortical bone [7,8,9,10,11,12,13].

On the other hand, to achieve a good osseointegration, implant surface should promote adhesion, proliferation, and differentiation of bone tissue cells. At the same time, it is highly desirable to avoid adhesion and growth of bacteria at bone-implant interface, since this can cause infections and subsequent implant failure [14]. In order to improve osseointegration, many approaches have been studied, including implant chemical and physical surface modifications, the most usual procedure. Chemical techniques comprise the introduction of natural or artificial chemical compounds, with elements that favor the interaction between implant and bone tissue cells, promoting bone ingrowth on the implant’s surface. In general, chemical techniques include coating, impregnation, immersion, or deposition of bioactive materials, such as hydroxyapatite, bioglasses, ceramics, polymers, or peptides [15,16,17], onto the surface. Physical methods consist of techniques focused on modifying the surface topography, altering its porosity, roughness, or smoothness. Some examples of physical modifications are sand- and grit-blasting, acid-etching, plasma-spraying, laser surface modification [14], ultraviolet treatment, electrochemical, and oxidation (anodization) methods [1]. Physical modifications of titanium and alloy implant surfaces could allow the creation of micro- and nanostructures to stimulate osseointegration [18,19] by increasing: porosity for cell adhesion and proliferation, as well as roughness to enhance wettability for protein adsorption, or smoothing surface for repelling bacterial infection.

Among the latest physical techniques, femtosecond laser ablation stands out as very advantageous due to its accurate control of designed features on the surface, its high efficiency, and low material consumption [1]. In Vorobyev and Guo’s pioneering work [18] on titanium substrates, the use of femtosecond laser ablation allowed the creation of nano- (pores, spherical protrusions, and multiple grooved surface patterns) and micro-structures (such as varied roughness configurations and smooth surface with micro-inhomogeneities) with appropriate adjustment of laser parameters. Recently, Rodríguez et al. [14] studied the influence of femtosecond laser modifications performed in porous titanium discs, producing a hierarchical arrangement composed of micro-holes, micro-columns, and a periodic surface nanometric structure, both on the flat surface and inside the pores. These modifications boosted superficial porosity and roughness, without any significantly affected mechanical properties of the titanium samples. Moreover, other authors have performed in vitro experiments on porous titanium substrates treated with femtosecond laser ablation, showing improved cell viability, as well as better differentiation morphology and cell adhesions, with acceptable biological response [20].

Femtosecond laser surface modifications on titanium and alloys substrates have been proved as a feasible tool to improve cells adhesion, differentiation, proliferation, and all together, more effectively boost osseointegration of the implant. In general, this physical modification technique allows: (1) custom design of nano- and micro-structures, such as laser induced periodically surface structure (LIPSS), ripples, columns, pits, and spikes [21,22,23], with an appropriate selection of laser beam parameters and conditions [24,25,26,27]; (2) formation of roughness with enhancement of wettability [28,29] or hydrophilicity-hydrophobicity of treated surfaces [29,30]; (3) inducing protein adsorption and following localized adhesion formation and cell shape-based mechanical restraints that promote osteogenic differentiation and hence, superior osseointegration of implants [31,32]; (4) prevention of bacterial adhesion and biofilm formation [33,34]; (5) variation of chemical composition of laser modified surfaces, for instance, bone-like apatite precipitation [35,36] and formation of nano- or micro-layers of oxides [37] such as, for example, protective TiO_2_ on titanium substrates.

The increasing demand of titanium and its alloys, as medical implants, requires a practical technique to control evolution in time of cells adhesion, proliferation, and differentiation, meaning the osseointegration process. Furthermore, it is also required to check implant surface features in the exposed biological surrounding, such as corrosion resistance, ions migration, durability, etc. It is, therefore, that researchers have thoroughly used Electric Impedance Spectroscopy (EIS) as an electrochemical tool for both purposes. In this sense, several corrosion studies have been carried out on titanium or titanium alloys surfaces, using electrochemical impedance spectroscopy [38,39,40] to follow the formation of passive TiO_2_ layer. Moreover, other surface features could be evaluated using impedance spectroscopy, such as porosity or pores sizes, with a clear advantage over other techniques such as Image Analysis or Archimedes Test, since it is a non-invasive technique and enables measurements to be carried out in situ. For example, Olmo et al. [41] used EIS for the characterization of porous titanium substrates, obtaining superior differences of total porosities, higher frequencies measured at electrical impedances, being 355–500 µm range of pore size and more sensitive to slight variations in impedance. Similarly, Chen et al. showed the negative effect of pores in corrosion resistance and higher corrosive rates in the presence of flowing electrolyte [42].

In recent years, Electrochemical Impedance Spectroscopy has become a leading topic for monitoring the evolution of cells adhesion, differentiation, and proliferation. Many studies have been led by researchers in this field, so it could be highlighted that Giner et al. work [43] performed in-situ evaluation of osteoblast cells growth on porous titanium substrates, studying the biological response of MC3T3E1, a murine pre-osteoblast cell line, by analysis of viability, morphology, differentiation, and alkaline phosphatase activity. Huang et al. showed that Electrochemical Impedance Spectroscopy could be used for in vivo measurement of U-2 OS osteoblast-like cell adhesion, spreading, and proliferation stage, on titanium and Ti-6A-4V implants, proposing equivalent circuits for each system [44]. An outstanding study was done by Nodberg et al., showing the suitability of electrical impedance spectroscopy to monitor, in real time, osteogenic differentiation of human Adipose Stem Cells (hASCs) of age-grouped donors, resulting in distinctive complex impedance patterns for each age group of cells [45]. Besides, Hamal et al. have summarized a wide range of Electrical Impedance measurements in cellular assays and its usefulness in regenerative medicine [46].

The present work is focused on the assessment of femtosecond laser modified porous titanium substrates using Electrical Impedance Spectroscopy. The objective is to analyze the impedance response due to diverse types of surface topographies, as different pores and pore sizes, total porosities, and oxide layers generated by femtosecond laser treatment (FT). Furthermore, osteoblast cells adhesion, differentiation, and proliferation will be monitored by electrical impedance measurements in previously modified titanium substrates. The aim of this study is to validate the utility and high sensitivity of the Electrical Impedance Spectroscopy technique to detect and differentiate subtle surface changes and its direct influence on osteoblast cells responses.

## 2. Materials and Methods

### 2.1. Manufacturing of Surface Modified Porous Titanium Discs Using Femtosecond Laser Surface Treatment

All samples were manufactured according to a methodology previously published [14,20,41,43]. Fully-dense commercially pure titanium (c.p. Ti–Grade IV, SEJONG Materials Co., Ltd., Incheon, Korea) discs were prepared using conventional Powder Metallurgy Technology, by pressing and sintering at 1300 MPa and 1250 °C, respectively. Meanwhile, porous titanium samples were manufactured by space holder technique, with a particle size range of 100–200 μm. Ammonium hydrogen carbonate (NH_4_HCO_3_) from (Cymit Química SL, Barcelona, Spain) was used as a space holder with different content (30, 40, 50, and 60 vol. %). Subsequently, the mixture of titanium powder and spacer particles were pressed at 800 MPa and then, spacer was removed using a low vacuum furnace (Heraeus, Hanau, Germany) (10^−2^ mbar) in two steps (60 °C and 110 °C) during 10 h each, and sintered at 1250 °C in a molybdenum chamber furnace (Termolab-Fornos Eléctricos, Lda., Agueda, Portugal) under high vacuum atmosphere (~10^−5^ mbar) for 2 h. The surface of the discs, with 12 mm in diameter and approximately 5 mm high, were polished with magnesium oxide (MgO) and hydrogen peroxide (H_2_O_2_) prior any surface treatments using femtosecond.

Femtosecond laser irradiation was performed following the methodology presented by Rodriguez [14] and Trueba [20] and collaborators, using a Yb-doped fiber laser (Spirit 1040-4, Spectra-Physics, Santa Clara, CA, USA) with a wavelength of 1040 nm and pulses of 396 fs, at a repetition rate of f = 100 kHz. A pulse energy of Ep = 49.7 μJ (100% of nominal power) and a scanning speed of v = 960 mm/s were chosen. After deflection by a galvanometer scanner, the laser beam was focused through an F-Theta lens (f = 160 mm) to a beam radius of approximately w0 = 12 μm on the working surface. The resulting laser fluency on the surface was F = 21.98 J/cm^2^. The surface of the samples was scanned line by line with the moving laser beam, and the laser paths were separated from each other according to an overlap of s = 50%. The surface was processed multiple times (Nr = 20), to increase the energy deposited on the surface. Under these conditions, the resulting number of pulses per point (PPS) at the surface was PPS = 100. The experiments were performed in air, and Argon was used as shielding gas in order to reduce any undesirable oxidation on the surface of the workpiece.

**Figure 1:**
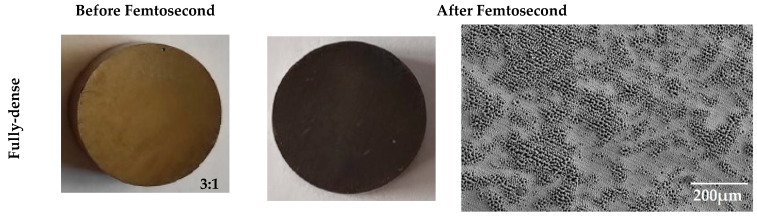

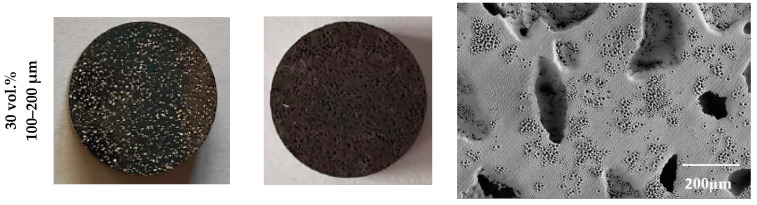
Optical and SEM images of some of the samples studied, before and after FS. All optical images are on the same scale, 3:1 (mm). FD: fully dense sample; 30 vol. %: sample with 30% porosity volume.

Macrostructure (high resolution Nikon camera) and microstructure (by scanning electron microscopy, using a Zeiss EVO LS 15 scanning electron microscope (Zeiss, Oberkochen, Germany) with an acceleration voltage of 10 kV) of two types of discs fabricated: fully dense (FD) and 30% porosity volume, taken before and after the treatment with femtosecond laser, is shown in Figure 1. The laser surface treatment was similar to the one performed in [14], but it was applied over a greater range of total porosity percentages. The resulting surface morphology is mostly independent of the volume of porosity of the samples and, therefore, the results of the laser treatment are similar to those presented in the previously published work. The surface, on the one hand, consists of macro pores generated by the spacers, the size and quantity of which depends on the total volume of the spacer. The laser treatment, on the other hand, generates, on the surface, a multiscale hierarchical texture. This texture is based on a mixture of clusters of micropores and micro-pillars, with characteristic lengths less than 10 μm, as well as laser-induced periodic surface structures (LIPSS), which are self-organized periodic nanostructures that cover the entire surface. These nanoripples are aligned perpendicularly to the polarization of the laser beam, and the spatial period of the structure is close to the wavelength of the laser (1040 nm).

### 2.2. Electrical Impedance Characterization

As previously presented, the role of porosity to solve the stress-shielding phenomenon (mismatch of young modulus between the implant-cortical bone) guarantees the bone-ingrowth as well as allows infiltration and adhesion of the coatings, with it being widely recognized in the scientific literature. The improvement of osseointegration capacity is associated with the surface roughness patterns obtained with a femtosecond laser radiation [31,32,33,35,36]. In previous works, the authors have used the Archimedean method, image analysis, Micro-CT, and scanning electron microscopy to evaluate the macro and microporosity of porous titanium samples, with and without surface modification [12,17,19,20]. However, the experimental protocols, commonly used to characterize porosity and evaluate cell activity (presence of osteoblastic cells and mineralization), are relatively long, expensive, and destructive. In this work, the use of electrical impedance is proposed, not only as an interesting route to semi-quantitatively evaluate porosity but also as a potential changes inherent to surface modification treatments. They can be used to improve osseointegration as well as to detect, in real time, the changes that may occur in the implant/bone interface, during this process.

**Figure 2:**
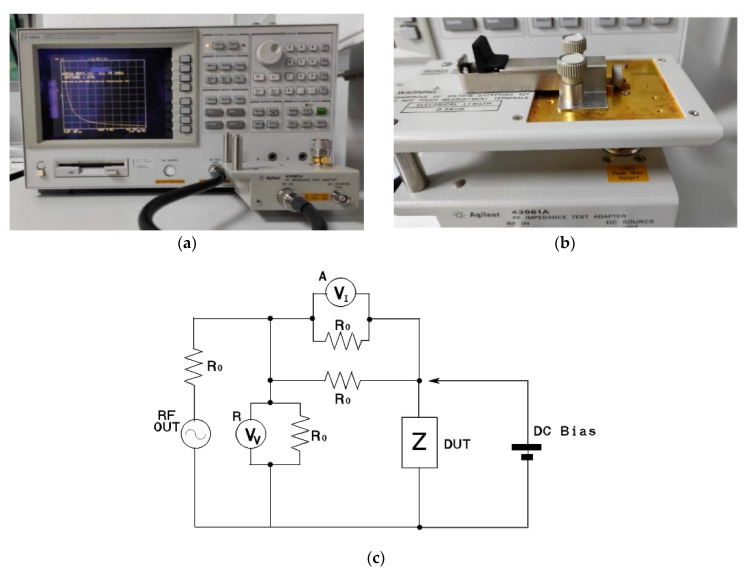
(**a**) impedance analyzer used (Hewlett–Packard 4395A). (**b**) placement of the sample in the HP 16,092 module. (**c**) measurement circuit used by the impedance analyzer, where the source signal is output from RF OUT port. Vv voltmeter is R port receiver that measures a voltage. Vi voltmeter is A port receiver that measures a voltage of Ro to obtain a current.

Hewlett–Packard 4395A (Agilent Technologies, Santa Clara, CA, USA), a network, spectrum, and impedance analyzer, available at IMSE-CNM-CSIC, was the equipment used to perform the electrical impedance measurements, as it is demonstrated in Figure 2a. Impedance measurements represent an affordable method to characterize, in a non-destructive way, different materials, while being especially useful to characterize surface modifications, as shown in different works [41]. To place the manufactured titanium samples on the impedance analyzer, the module HP 16092A was used, as Figure 2b indicates. Figure 2c also proves the implemented circuit by the impedance analyzer, where the sample is placed in the DUT (device under test).

Electrical impedance was measured in the frequency range from 150 MHz to 500 MHz. These measurements were performed three times for samples: before a femtosecond laser treatment, after a femtosecond laser treatment (FS), and with cell cultures (CC). Afterwards, the pore content, the effect of the femtosecond laser treatment [potential oxide layer, new microporosity (pillars), and new additional surface area (generated by the new texture of the roughness pattern)] were evaluated.

In-vitro cellular behavior (adhesion and proliferation of osteoblasts) in fully-dense and porous discs (before and after femtosecond treatment) was also evaluated. To get it, MC3T3E1, a murine pre-osteoblast cell line (CRL-2593, from ATCC), was used. Cell proliferation and viability tests were evaluated using AlamarBlue^®^ reagent (Invitrogen, Carlsbad, CA, USA), in accordance with the manufacturer’s protocol. The absorbance at 570 nm (oxidized) and 600 nm (reduced) (TECAN, Infinity 200 Pro, Männedorf, Switzerland) was subsequently recorded, and these experiments were performed in triplicate. The results were expressed in terms of mean and standard deviation to perform two-way ANOVA, followed by Tukey’s post-test, using SPSS v.22.0 for Windows (IBM Corp., Armonk, NY, USA). The significance level was considered at p values of *p* ˂ 0.05 (*).

Additionally, cell behavior at 21 days was evaluated by the acquisition of images with a scanning electron microscopy (SEM) (Zeiss EVO LS 15 scanning electron microscope) (Zeiss, Oberkochen, Germany). Once the osteoblast cells were grown along the surface of the discs, electrical impedance measurements were obtained again. In order to assess the effect of cell growth on the material and its electrical properties, this process uses the same equipment and configuration as the samples before femtosecond laser treatment and with femtosecond laser treatment (FS).

## 3. Results and Discussion

### 3.1. Electrical Characterization of Porous Discs and Femtosecond Laser Treatment

**Figure 3:**
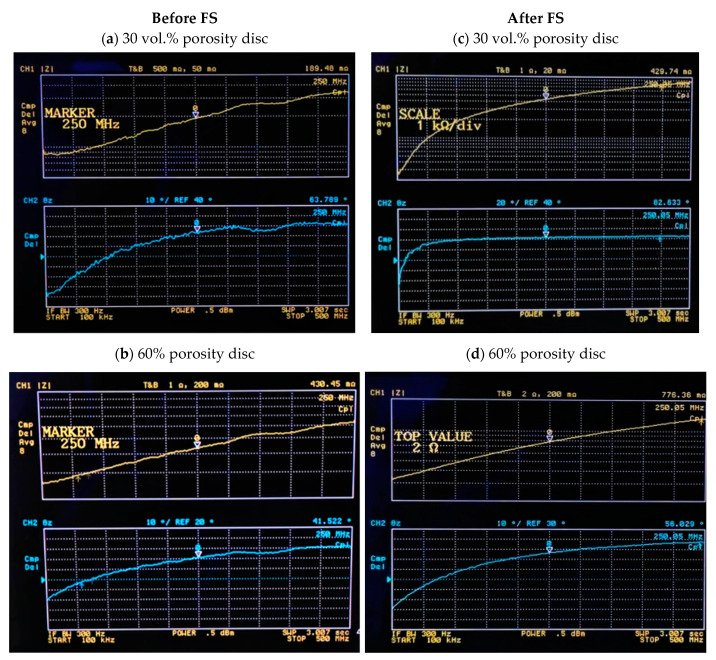
Graphs obtained with the modulus and phase impedance of titanium samples, with 30% and 60% porosity volume. (**a**) sample with 30% porosity volume before FS, (**b**) sample with 60% porosity volume before FS, (**c**) sample with 30% porosity volume after FS, and (**d**) sample with 60% porosity volume after FS.

Figure 3 shows, as an example, the graphs obtained for an impedance value of samples, with 30% and 60% porosity volume, before and after femtosecond laser treatment. As can be appreciated in each image, the upper graph in yellow, corresponding to channel 1, represents the modulus |Z| in mΩ of the obtained impedance. The second graph in the color blue, corresponding to channel 2, represents the phase θz of the impedance in degrees. In this figure, it can be observed that the marker is at 250 MHz, and it shows the impedance and phase corresponding to that frequency. It allows one to see how the modulus impedance value increases with the percentage of porosity volume and with the femtosecond laser treatment.

**Figure 4:**
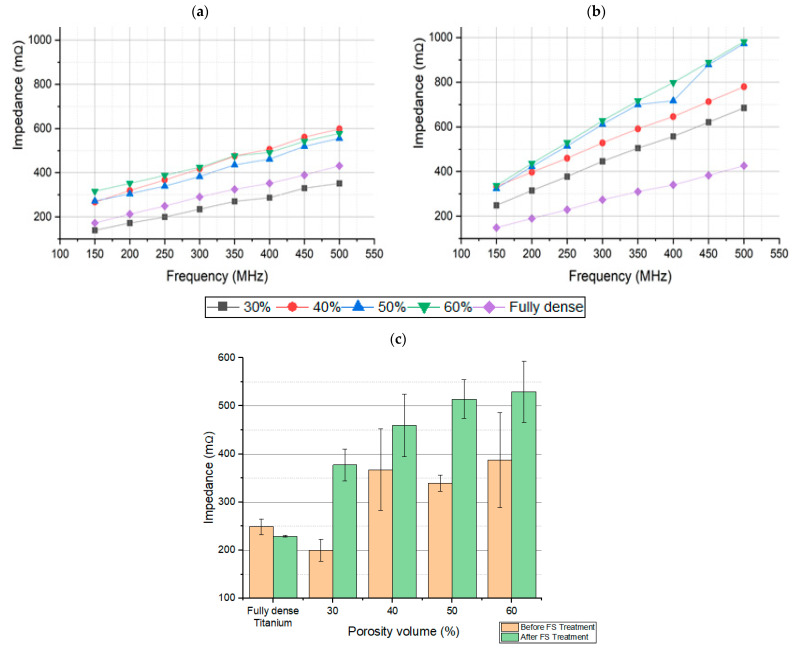
Impedance of the different samples with pore size 100–200 μm (**a**) before FS, And (**b**) after FS. The impedance values are directly proportional to the frequency, with higher sensitivities at higher frequencies. (**c**) Impedance values vs porosity volumes at 250 MHz.

The absolute value of impedance of the different samples, before and after the application of the laser, at the range of frequencies studied, is proven in Figure 4. It can be seen how the impedance values increase directly proportional to frequency, as expected, and in relation with other previous studies [41]. It is distinguished for a specific size of pore, by different samples with different porosity values. It is also observed that more sensitivity is obtained at higher frequencies, i.e., porosity volume can be better distinguished at higher frequencies, while still in relation with previous studies [41].

Laser application produces an important increase in the absolute value of the impedance (Figure 4b). In Figure 4c, the dispersion of measurements at the specific frequency of 250 MHz is observed. It is also shown the importance of an increase in the absolute values of the impedance of the sample after the treatment with the femtosecond laser, in comparison with the samples without laser treatment. These results can be explained by the change on the surface, which was produced by the laser treatment.

The values of the electrical impedance modulus, together with the values of the phase (imaginary part of the impedance), are shown for three different frequencies in Table 1, for the samples before FS. With these measurements, the relationship between the increase in electrical impedance and the increase in porosity can be seen.

Similarly, for the case of the samples after FS, data were collected at three different frequencies in Table 2. It shows the increase in the electrical impedance values with the increase in the porosity percentage. Moreover, these values have increased with respect to those obtained for the samples before FS.

### 3.2. Electrical Characterization of Osteoblast Cell Cultures: Cell Proliferation and Viability Tests

**Figure 5:**
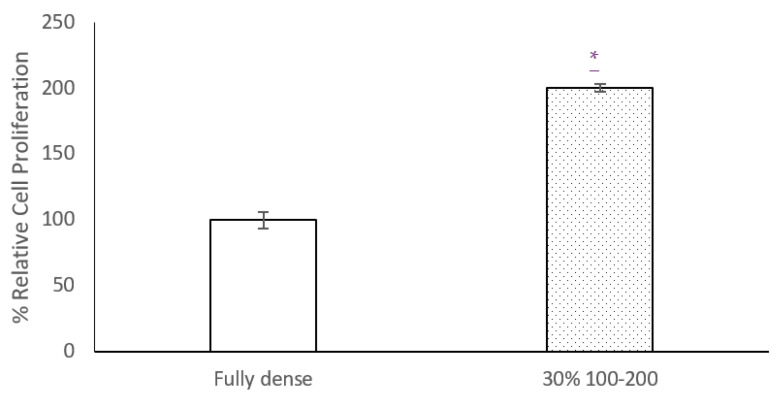
Percentage relative cell proliferation in MC3T3–E1 cultures after femtosecond laser treatment (by AlamarBlue). Results are represented vs fully dense growth. Significance level at *p* value < 0.05 (*).

Cell proliferation results (Figure 5) prove that there is better osteoblastic growth on the surface of samples with 30% porosity than on fully dense samples. It is observed that, in the 30% discs, the proliferation % is double compared to FD, making the increase statistically significant.

**Figure 6:**
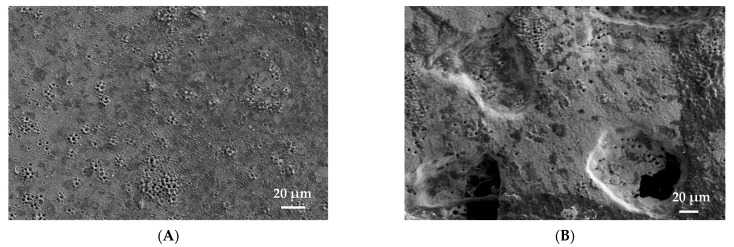
SEM Image of a samples of porous titanium after the femtosecond surface treatment in-vitro culture (observe the presence of adhered osteoblasts). (**A**) Fully-dense; (**B**) 30 vol. % (100–200 µm).

**Figure 7:**
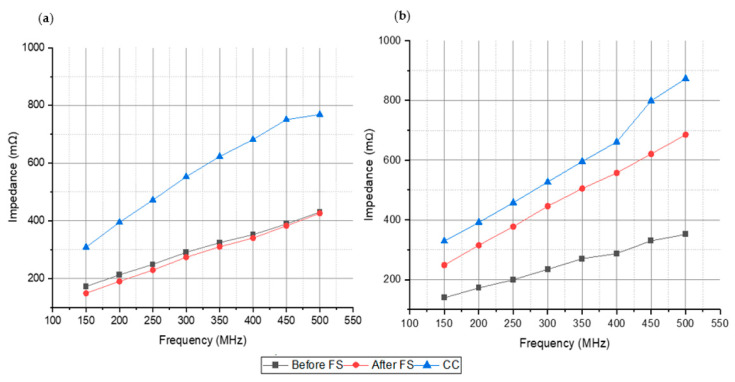
(**a**) Impedance values of discs with cell cultures (CC) vs previous impedance values before FS and after FS in fully dense sample. (**b**) impedance values in 30% porosity volume sample.

Figure 6 also shows, as an example, a SEM image of adhered cells on a porous titanium surface modified with laser radiation, where the in-vitro osseointegration can be verified. These results correlate with the measured impedance of the samples, especially at higher frequencies, where higher impedance is measured for the 30% samples. On the other hand, Figure 7a,b show the absolute values of impedance measurements in samples with osteoblast cell cultures. An increase in the absolute value of the measured impedance is observed repeatedly, in line with previous studies [43]. A comparison between samples without treatment, with femtosecond laser treatment, and samples with cell cultures is presented in the whole range of frequencies. In Figure 7a, the absolute value of fully dense samples with osteoblast cell cultures is higher for all frequencies to the impedance measurements for samples with femtosecond laser treatment and without it. Absolute values of impedance measurements for 30% porosity volume samples is reproduced in Figure 7b. Again, the highest values of impedance are observed for samples with cell cultures.

The increase in the absolute value of the impedance due to the osteoblast cell growth is, in all cases, higher than the increase due to the femtosecond laser treatment. This increase suggests that the capacitive layer, formed by the cell membranes on the disc’s surface, has a more relevant effect on the overall impedance. Therefore, both cases can also be distinguished with electrical impedance in an affordable, non-destructive, and simple way (Table 3).

The experimental protocols commonly used to characterize porosity and evaluate cell activity (presence of osteoblastic cells and mineralization) are relatively long, expensive, and destructive. To avoid the mentioned issues, an electrical impedance measurement protocol is proposed as an alternative. This simple protocol makes it possible to evaluate the pore content of a material and to detect physical and chemical changes that may occur on the surface of implants; resulting from surface modification treatments and the interaction of the implant with the surrounding tissue (osseointegration process). In this context, a clear direct relation is observed between electrical impedance and pore content, femtosecond laser treatment and cellular activity (adhesion and proliferation of osteoblasts).

Electrical impedance spectroscopy was used for the characterization of different porous titanium samples, modified with a femtosecond laser. Different volumes of porosities could be distinguished, in line with previous studies. The treatment of the samples with the femtosecond laser produced a significant increase in the absolute value of the electrical impedance, which can be perfectly characterized in a wide range of frequencies. It made them be more sensitive at higher frequencies. Furthermore, the in-vitro cellular behavior (adhesion and proliferation of osteoblasts) in porous discs was also evaluated, and an increase in the absolute values of the impedance was observed for all titanium samples where cells were cultivated, according to previous works. This increase in the impedance values was higher, in all cases, than the increase in the impedance produced by the treatment with femtosecond laser, for all the tested samples.

## 4. Conclusions

The method followed in this study has proven to be effective for the characterization of the treatment of the surface topography of titanium implants with femtosecond laser, through electrical impedance measurements. It has also demonstrated to be a valid tool in the study of subsequent osseointegration processes, with the characterization of the growth of osteoblast cell cultures in the same samples.

Significant differences can be appreciated in the impedance values obtained for the samples at different percentages of porosity volume and the fully dense sample. This occurs for the samples before FS treatment and after FS treatment, being higher frequencies (around 500 MHz) and the ones that show a better sensitivity to impedance changes. For the samples treated with femtosecond laser, a huge increase (higher than 50% in some cases) in the electrical impedance values is observed, compared to the values obtained for the untreated samples. This fact shows that the modification of the surfaces of the samples favors the increase in the electrical impedance.

In addition, it has also been observed that the cell culture (MC3T3-E1) influences the electrical impedance values obtained for the samples. The effect of the increase in electrical impedance is greater in the fully dense samples, where an increase higher than 70% is found at 500 MHz.

As a future work, it would be interesting to design a bioimpedance device that allowed real-time measurements while the cells are growing on the implant sample inside the incubator. This would enable one to obtain interesting data of the process of osseointegration, in the implant, in order to study possible future uses of the technique in medical applications.

## Figures and Tables

**Table 1:** Impedance modulus and phase values for different titanium samples with pore size 100–200 μm before FS.

Frequency	Fully-Dense Titanium		30 vol.%		40 vol.%		50 vol.%		60 vol.%	
	|Z| (mΩ)	θ	|Z| (mΩ)	θ	|Z| (mΩ)	θ	|Z| (mΩ)	θ	|Z| (mΩ)	θ
150 MHz	172.23	58.78°	139.32	52.88°	266.70	51.12°	271.96	48.83°	315.43	35.42°
250 MHz	249.02	66.85°	199.57	65.72°	367.48	56.82°	339.57	58.15°	387.5	44.93°
500 MHz	430.92	75.01°	351.56	72.24°	598.41	59.71°	555.33	65.44°	577.31	52.74°

Note: Impedance measurements have an error of ±0.1. |Z| is the impedance modulus measured in milliohms (mΩ), and θ is the phase measured in degrees.

**Table 2:** Impedance modulus and phase values for different titanium samples with pore size 100–200 μm after FS.

Frequency	Fully-Dense Titanium		30 vol.%		40 vol.%		50 vol.%		60 vol.%	
	|Z| (mΩ)	θ	|Z| (mΩ)	θ	|Z| (mΩ)	θ	|Z| (mΩ)	θ	|Z| (mΩ)	θ
150 MHz	148.81	63.37°	248.74	63.03°	333.19	53.33°	324.02	74.84°	337.07	72.66°
250 MHz	229.25	73.21°	377.33	69.02°	459.78	61.16°	514.23	78.37°	529.88	75.58°
500 MHz	426.06	79.17°	685.04	73.85°	779.71	70.5°	972.62	81.70°	981.51	77.34°

Note: Impedance measurements have an error of ±0.1. |Z| is the impedance modulus measured in milliohms (mΩ), and θ is the phase measured in degrees.

**Table 3:** Impedance modulus and phase values for different titanium samples with osteoblast cell cultures (CC).

Frequency	Fully-Dense Titanium		30 vol.%	
	|Z| (mΩ)	θ	|Z| (mΩ)	Θ
150 MHz	308.6	67.55°	328.967	50.62°
250 MHz	472.01	68.19°	457.557	63.71°
500 MHz	768.385	67.24°	873.023	74.54°

Note: Impedance measurements have an error of ±0.1.

## Data Availability

The data presented in this study are available on request from the corresponding author.
